# Ventilatory Assistance Before Umbilical Cord Clamping in Extremely Preterm Infants: A Randomized Clinical Trial

**DOI:** 10.1001/jamanetworkopen.2024.11140

**Published:** 2024-05-17

**Authors:** Karen D. Fairchild, Gina R. Petroni, Nikole E. Varhegyi, Marya L. Strand, Justin B. Josephsen, Susan Niermeyer, James S. Barry, Jamie B. Warren, Monica Rincon, Jennifer L. Fang, Sumesh P. Thomas, Colm P. Travers, Andrea F. Kane, Waldemar A. Carlo, Bobbi J. Byrne, Mark A. Underwood, Francis R. Poulain, Brenda H. Law, Terri E. Gorman, Tina A. Leone, Dorothy I. Bulas, Monica Epelman, Beth M. Kline-Fath, Christian A. Chisholm, John Kattwinkel

**Affiliations:** 1 Division of Neonatology, Department of Pediatrics, University of Virginia, Charlottesville; 2 Division of Translational Research and Applied Statistics, Department of Public Health Sciences, University of Virginia, Charlottesville; 3 Division of Neonatology, Department of Pediatrics, St Louis University, St Louis, Missouri; 4 Section of Neonatology, Department of Pediatrics, University of Colorado, Denver; 5 Division of Neonatology, Department of Pediatrics, Oregon Health & Science University, Portland; 6 Division of Maternal-Fetal Medicine, Department of Obstetrics and Gynecology, Oregon Health & Science University, Portland; 7 Division of Neonatal Medicine, Department of Pediatric and Adolescent Medicine, Mayo Clinic, Rochester, Minnesota; 8 Section of Newborn Critical Care, Department of Pediatrics, University of Calgary, Alberta, Canada; 9 Division of Neonatology, Department of Pediatrics, University of Alabama at Birmingham; 10 Division of Neonatology, Department of Pediatrics, Indiana University, Indianapolis; 11 Division of Neonatology, Department of Pediatrics, University of California, Davis, Sacramento; 12 Division of Neonatology, Department of Pediatrics, University of Alberta, Edmonton, Canada; 13 Division of Neonatology, Department of Pediatrics, Brigham and Women’s Hospital, Boston, Massachusetts; 14 Division of Neonatology, Department of Pediatrics, Columbia University, New York, New York; 15 Department of Radiology, Children’s National Medical Center, Washington, DC; 16 Department of Radiology, Nemours Children’s Hospital, Orlando, Florida; 17 Department of Radiology, Cincinnati Children’s Hospital Medical Center, Cincinnati, Ohio; 18 Division of Maternal-Fetal Medicine, Department of Obstetrics and Gynecology, University of Virginia, Charlottesville

## Abstract

**Importance:**

Providing assisted ventilation during delayed umbilical cord clamping may improve outcomes for extremely preterm infants.

**Objective:**

To determine whether assisted ventilation in extremely preterm infants (23 0/7 to 28 6/7 weeks’ gestational age [GA]) followed by cord clamping reduces intraventricular hemorrhage (IVH) or early death.

**Design, Setting, and Participants:**

This phase 3, 1:1, parallel-stratified randomized clinical trial conducted at 12 perinatal centers across the US and Canada from September 2, 2016, through February 21, 2023, assessed IVH and early death outcomes of extremely preterm infants randomized to receive 120 seconds of assisted ventilation followed by cord clamping vs delayed cord clamping for 30 to 60 seconds with ventilatory assistance afterward. Two analysis cohorts, not breathing well and breathing well, were specified a priori based on assessment of breathing 30 seconds after birth.

**Intervention:**

After birth, all infants received stimulation and suctioning if needed. From 30 to 120 seconds, infants randomized to the intervention received continuous positive airway pressure if breathing well or positive-pressure ventilation if not, with cord clamping at 120 seconds. Control infants received 30 to 60 seconds of delayed cord clamping followed by standard resuscitation.

**Main Outcomes and Measures:**

The primary outcome was any grade IVH on head ultrasonography or death before day 7. Interpretation by site radiologists was confirmed by independent radiologists, all masked to study group. To estimate the association between study group and outcome, data were analyzed using the stratified Cochran-Mantel-Haenszel test for relative risk (RR), with associations summarized by point estimates and 95% CIs.

**Results:**

Of 1110 women who consented to participate, 548 were randomized and delivered infants at GA less than 29 weeks. A total of 570 eligible infants were enrolled (median [IQR] GA, 26.6 [24.9-27.7] weeks; 297 male [52.1%]). Intraventricular hemorrhage or death occurred in 34.9% (97 of 278) of infants in the intervention group and 32.5% (95 of 292) in the control group (adjusted RR, 1.02; 95% CI, 0.81-1.27). In the prespecified not-breathing-well cohort (47.5% [271 of 570]; median [IQR] GA, 26.0 [24.7-27.4] weeks; 152 male [56.1%]), IVH or death occurred in 38.7% (58 of 150) of infants in the intervention group and 43.0% (52 of 121) in the control group (RR, 0.91; 95% CI, 0.68-1.21). There was no evidence of differences in death, severe brain injury, or major morbidities between the intervention and control groups in either breathing cohort.

**Conclusions and Relevance:**

This study did not show that providing assisted ventilation before cord clamping in extremely preterm infants reduces IVH or early death. Additional study around the feasibility, safety, and efficacy of assisted ventilation before cord clamping may provide additional insight.

**Trial Registration:**

ClinicalTrials.gov Identifier: NCT02742454

## Key Points

**Question:**

For extremely preterm infants, does providing ventilatory assistance during 120 seconds of delayed umbilical cord clamping, compared with 30 to 60 seconds of delayed cord clamping with ventilatory assistance after, reduce intraventricular hemorrhage (IVH) or early death?

**Findings:**

In this randomized clinical trial of 570 infants born at less than 29 weeks’ gestation, no difference was detected in the primary outcome of IVH on 7- to 10-day head ultrasonography or death before day 7.

**Meaning:**

In this study, assisting ventilation prior to cord clamping did not reduce IVH or death compared with cord clamping followed by standard resuscitation; further study may provide insight into the feasibility, safety, and efficacy of assisted ventilation before cord clamping for other outcomes.

## Teaser

This randomized clinical trial assesses whether assisted ventilation in extremely preterm infants before umbilical cord clamping reduces intraventricular hemorrhage or early death.

## Introduction

Effective cardiorespiratory transition from fetal to neonatal life requires lung inflation, increased pulmonary blood flow, and increased oxygenation.^1^ In preterm animal models, clamping the umbilical cord before lung inflation results in fluctuations in cerebral blood flow and oxygenation,^2,3,4^ conditions that may increase risk of intraventricular hemorrhage (IVH) in human infants born extremely preterm (gestational age [GA] of 23 0/7 to 28 6/7 weeks).

The American College of Obstetricians and Gynecologists supports delay of umbilical cord clamping for 30 to 60 seconds after preterm delivery based on evidence of improved survival and other benefits.^5,6,7^ An early meta-analysis of randomized clinical trials of delay for at least 30 seconds compared with immediate cord clamping for preterm infants reported a significantly lower risk of any grade of IVH.^5^ A 2023 individual participant data meta-analysis reported that deferring cord clamping for increasingly longer periods was associated with progressively lower mortality.^8^ Importantly, however, infants not breathing well were excluded from many of these trials.^9^ Several small clinical trials have demonstrated feasibility of providing ventilatory assistance to preterm infants with intact placental circulation without identified safety concerns.^10,11,12,13^

The aim of this trial was to test the hypothesis that providing assisted ventilation starting 30 seconds after birth and clamping the cord at 120 seconds, compared with delaying cord clamping for 30 to 60 seconds with breathing assistance given afterward, would reduce any grade of IVH on head ultrasonography 7 to 10 days after birth or the competing outcome of death before day 7 in extremely preterm infants. We hypothesized that infants not breathing well would derive the most benefit from assisted ventilation; therefore, we planned a priori to study 2 cohorts of infants separately based on whether they were breathing well within 30 seconds after birth.

## Methods

### Study Design

VentFirst was a phase 3, 1:1, parallel-stratified randomized clinical trial conducted at 12 centers in the US and Canada. The trial protocol is provided in Supplement 1. The institutional review board of each center approved the protocol, and written informed consent from the mother was required prior to delivery. The consent form was available in English and Spanish. An optional video was available in English and Spanish to supplement the informed consent process.^14^ This study followed the Consolidated Standards of Reporting Trials (CONSORT) reporting guideline.

Randomization occurred before delivery. Control group infants received delayed cord clamping of 30 to 60 seconds after birth followed by assisted ventilation, and intervention group infants received assisted ventilation from 30 until 120 seconds after birth followed by cord clamping. Two analysis cohorts were identified a priori: infants not breathing well within 30 seconds after birth and those breathing well. Centers began by enrolling women with singleton pregnancies. Once personnel had experience with the intervention, women with dichorionic twin pregnancies were eligible for enrollment. A data safety and monitoring committee (DSMC) reviewed adverse events, safety stopping bounds, balance of accrual to each group, and results from a prespecified single interim analysis.

### Inclusion and Exclusion Criteria

Enrollment occurred between September 2, 2016, and February 21, 2023. Women expected to deliver extremely preterm infants at 23 0/7 to 28 6/7 weeks’ gestation by best obstetric estimate were eligible for inclusion. Self-identified race (categorized as Asian, Black, White, other [American Indian or Alaska Native], multiracial, or not reported) and ethnicities (Hispanic or Latino or not Hispanic or Latino) data were collected for descriptive purposes and not further analyzed. Exclusions included monochorionic twin and higher-order multiple pregnancies, medical emergencies requiring immediate delivery, known major fetal anomalies, severe fetal anemia, hydrops fetalis, or decision not to pursue intensive care for the infant. In addition, the responsible obstetrician or neonatologist could exclude the participants due to other concerns about mother or fetus.

### Randomization

When delivery at less than 29 weeks’ gestation was thought to be imminent, randomization was performed using a computer-generated code and a stratified block randomization scheme with varying block sizes of 2 to 6. There were 2 stratification factors at randomization, study site and GA (23 0/7 to 25 6/7 weeks or 26 0/7 to 28 6/7 weeks). The randomization unit was the mother; therefore, twins were assigned to the same intervention.

### Protocol

For each delivery, a facilitator read the protocol script, asked for assessment of breathing at 30 seconds, announced cord clamping time based on study group, and documented infant status and interventions. If the responsible obstetric or neonatal clinician had concerns about the mother or infant, cord clamping could be performed prior to the designated time. Umbilical cord milking was not permitted, and oxytocin was not administered to the mother until after cord clamping.

In both study groups, initial steps of infant resuscitation included tactile stimulation and suctioning the airway, if needed. For infants randomized to the intervention arm, 1 or 2 neonatology clinicians trained in the protocol were assigned to perform the intervention. Immediately after delivery, infants were placed near the perineum for vaginal birth, on sterile-wrapped trays across the mother’s thighs for cesarean birth, or on freestanding platforms^15^ for initial steps of stabilization. Warming pads and plastic wrap were used to minimize heat loss. Thirty seconds after birth, the infant received continuous positive airway pressure if breathing well or positive pressure ventilation (PPV) if not breathing well. Heart rate was checked at 60 and 90 seconds, and if less than 100 beats per minute, ventilation corrective actions were performed per the American Academy of Pediatrics Neonatal Resuscitation Program (seventh and eighth editions).^16^ The goal cord clamping time was 120 seconds after birth. Equipment used for the intervention varied by site and included face masks, devices to administer continuous positive airway pressure and PPV, tubing, and oxygen and air tanks to provide blended supplemental oxygen. For cesarean deliveries, equipment near the sterile field was sterilized, covered in sterile wrap, or removed from single-use packaging in a sterile fashion.

For infants randomized to the control group, the umbilical cord was clamped 30 seconds after birth if the infant was not breathing well (apneic or gasping) or delayed until up to 60 seconds if the infant was breathing well (audible crying or visible sustained respirations).

After umbilical cord clamping, infants in both study arms were transferred to the radiant warmer for continued stabilization, and all other care throughout the neonatal intensive care unit (NICU) stay was per unit guidelines. Randomized mothers were followed up until their hospital discharge. Infants were followed up until 36 weeks’ postmenstrual age (PMA), death, or hospital discharge, whichever came first. For infants transferred to another unit prior to discharge, outcome data at 36 weeks’ PMA were requested from the transfer hospital.

### Masking

Due to the nature of the study intervention, clinicians present for delivery could not be masked to the study arm. To reduce risk of bias, clinicians were instructed to not record timing of cord clamping or study arm in the medical record, and radiologists interpreting head ultrasonography results were masked to group assignment.

### Outcomes Assessment

The primary outcome was IVH or death and was defined as any grade of IVH on head ultrasound 7 to 10 days after birth or death before day 7. Head ultrasonography was interpreted routinely by board-certified site radiologists masked to the study arm using Papile criteria,^17^ with the highest grade on either side taken as the outcome. External interpretation of each infant’s study head ultrasonography was performed by 1 of 3 independent board-certified pediatric radiologists (D.I.B., M.E., and B.M.K.-F.) masked to study arm and to local interpretation. If the local and first independent interpretation differed in maximum grade of IVH, another independent radiologist reviewed the ultrasonography results. Agreement between 2 readers was required for the final interpretation of maximum IVH grade.

Secondary outcomes included Apgar score less than 5 at 5 minutes, lowest hematocrit in the first 24 hours, medication for low blood pressure within 24 hours of birth, number of red blood cell transfusions in the first 10 days, death prior to 36 weeks’ PMA, and severe brain injury. Severe brain injury was based on the reading of site radiologists and was defined as grade 3 to 4 IVH, cerebellar hemorrhage, or cystic periventricular leukomalacia on the 7- to 10-day or 36-week PMA head ultrasonography.

Additional outcomes included 1- and 5-minute Apgar scores, umbilical cord arterial blood gas pH, and delivery room intubation. Outcomes in the first 24 hours included infants’ temperature at the time of NICU admission; pneumothorax requiring intervention; lowest mean blood pressure; highest hematocrit (prior to transfusion); Score for Neonatal Acute Physiology–Perinatal Extension-II^18^; and administration of any fluid bolus, including crystalloid, colloid, or blood products. Data from the first 10 days after birth included surfactant administration, number of days on mechanical ventilation via an endotracheal tube, maximum serum bilirubin level, days of phototherapy, and blood culture–positive early-onset sepsis (before age 3 days). Outcomes through 36 weeks’ PMA included spontaneous intestinal perforation, necrotizing enterocolitis (Bell stage 2-3), blood culture–positive late-onset sepsis (after age 3 days), bronchopulmonary dysplasia (supplemental oxygen or positive pressure requirement at 36 weeks’ PMA), severe retinopathy of prematurity (stage 3 or requiring medical or surgical treatment), and all deaths through 36 weeks’ PMA. For mothers, adverse events reported include postpartum hemorrhage of 1000 mL or more within 24 hours of delivery, blood transfusion between delivery and discharge, retained placenta, and delivery-related infection prior to discharge.

### Statistical Analysis

Sample size estimation was based on the primary outcome in the cohort of infants not breathing well at 30 seconds, stratified by GA at randomization: 23 0/7 to 25 6/7 weeks and 26 0/7 to 28 6/7 weeks. Under the assumption that a minimum of 37% of infants (maximum, 50%) would be in the not-breathing-well cohort, accrual of 940 infants would yield at least 80% power (2-sided *P* = .05) to detect an odds ratio of 0.50 for intervention compared with control in the not-breathing-well cohort based on a Cochran-Mantel-Haenszel (CMH) test statistic. The calculations were based on primary outcome event rates of 55% and 26% within GA strata for a weighted overall rate of 37%, and on 38% and 62% of infant accruals expected to be in GA strata 23 0/7 to 25 6/7 and 26 0/7 to 28 6/7, respectively.

The primary analysis was based on intention to treat, including all eligible randomized infants analyzed according to the group to which they were originally assigned. Analyses were prespecified in the study protocol and the statistical analysis plan (Supplement 1). Under the guidance of the DSMC and before investigators were made aware of study results, modifications to the statistical analysis plan included reporting relative risk (RR) rather than odds ratio and clarifying secondary outcomes. All planned analyses were conducted within cohort and adjusted for GA. Additional unplanned analyses included examination of study outcomes for all infants combined and adjustment for sex in the combined cohort for the primary outcome. The per-protocol analysis was performed for multiple outcomes, adjusted for GA and by breathing cohort, and included all infants except those for whom the protocol could not be initiated or for whom cord clamping occurred at least 15 seconds from the protocol-prescribed time. Several sensitivity analyses were performed, including (1) assessment of potential bias of breathing cohort determination, which occurred after randomization; (2) adjustment for site (as well as GA); and (3) potential effect of correlation in outcomes between twins.

The primary and binary secondary infant outcomes were analyzed by the stratified CMH test for RR by study arm with effects summarized by point estimates and 95% CIs. Regression analysis consistent with the scale of the outcome measure was used for other secondary outcomes, including generalized linear regression models for continuous outcomes and negative binomial for count outcomes, all with independent variables for stratification factor (GA) and intervention assignment. Given the excess number of observed zeros in count of days receiving mechanical ventilation and number of blood transfusions, zero-inflated regression models were used to determine whether results varied. Missing data were limited and not imputed since the majority were missing due to infant death and multivariable modeling was not performed. To minimize the number of empty cells in CMH estimation for GA and site adjustment, sites with fewer than 25 enrolled infants were grouped as 1 site. Generalized estimating equations were used to account for any correlation in the primary outcome between twins. Preplanned statistical hypothesis testing was not performed since the study did not reach target accrual. Results were deemed of possible interest if the CIs around outcomes did not contain parameter equality. Statistical analyses were performed using SAS, version 9.4 software (SAS Institute Inc).

## Results

### Enrollment and Cohort Demographics

In total, 570 eligible infants were enrolled (median [IQR] GA, 26.6 [25.2-27.9] weeks, 273 females [47.9%] and 297 males [52.1%]; maternal race: 16 Asian [2.8%], 169 Black [29.7%], 340 White [60.0%], 3 other [0.5%], 8 multiracial [1.4%], and 34 not reported [6.0%]; maternal ethnicity: 64 Hispanic or Latino [11.2%], 478 not Hispanic or Latino [83.9%], and 28 [4.9%] not reported; and 44 twins [7.7%]) (Table 1). In contrast to the assumed 37% at study design, 47.5% (271 of 570) of enrolled infants were assessed as not breathing well 30 seconds after birth. A preplanned statistical analysis of the primary outcome was performed at two-thirds enrollment in the not-breathing-well cohort. Based on analysis results, the DSMC requested additional probability calculations of a positive outcome if accrual continued to target. Conditional probability calculations were generated under a wide range of possible odds ratios (change to RR was proposed by the DSMC after study closure) given the results to that point and if full target accrual was met. Results indicated that in the best-case scenario, there was less than 10% probability of rejecting the null hypothesis and recommended closure of the study.

**Table 1.** Infant Characteristics by Randomization Before Birth

Characteristic	No. (%)					
	Total		Not breathing well at 30 s		Breathing well at 30 s	
	Intervention (n = 278)	Control (n = 292)	Intervention (n = 150)	Control (n = 121)	Intervention (n = 128)	Control (n = 171)
GA stratum, wk						
23 0/7 to 25 6/7	116 (41.7)	115 (39.4)	71 (47.3)	61 (50.4)	45 (35.2)	54 (31.6)
26 0/7 to 28 6/7	162 (58.3)	177 (60.6)	79 (52.7)	60 (49.6)	83 (64.8)	117 (68.4)
GA at birth, median (IQR), wk	26.6 (24.9-27.7)	26.6 (25.4-27.9)	26.0 (24.4-27.6)	26.1 (24.7-27.4)	26.9 (25.6-28.0)	27 (25.9-28.0)
Birth weight, median (IQR), g	840 (650-1030)	813 (658-988)	760 (600-970)	750 (575-920)	885 (728-1065)	850 (708-1028)
Small for gestational age (<10th percentile)	40 (14.4)	60 (20.6)	27 (18.0)	32 (26.5)	13 (10.2)	28 (16.4)
Sex						
Female	132 (47.5)	141 (48.3)	65 (43.3)	54 (44.6)	67 (52.3)	87 (50.9)
Male	146 (52.5)	151 (51.7)	85 (56.7)	67 (55.4)	61 (47.7)	84 (49.1)
Maternal race						
Asian	9 (3.2)	7 (2.4)	0	2 (1.7)	9 (7.0)	5 (2.9)
Black	74 (26.6)	95 (32.5)	36 (24.0)	41 (33.9)	38 (29.7)	54 (31.6)
White	171 (61.5)	169 (57.9)	98 (65.3)	69 (57.0)	73 (57.0)	100 (58.5)
Other^a^	1 (0.4)	2 (0.7)	1 (0.7)	0	0	2 (1.2)
Multiracial	4 (1.4)	4 (1.4)	4 (2.7)	2 (1.7)	0	2 (1.2)
Not reported	19 (6.8)	15 (5.1)	11 (7.3)	7 (5.8)	8 (6.3)	8 (4.7)
Maternal ethnicity						
Hispanic or Latino	37 (13.3)	27 (9.3)	27 (18.0)	6 (5.0)	10 (7.8)	21 (12.3)
Not Hispanic or Latino	230 (82.7)	248 (84.9)	116 (77.3)	107 (88.4)	114 (89.1)	141 (82.5)
Not reported	11 (4.0)	17 (5.8)	7 (4.7)	8 (6.6)	4 (3.1)	9 (5.3)
Twin gestation	16 (5.8)	28 (9.6)	9 (6.0)	9 (7.4)	7 (5.5)	19 (11.1)
Preeclampsia	98 (35.3)	108 (37.0)	54 (36.0)	45 (37.2)	44 (34.4)	63 (36.8)
Preterm prelabor rupture of membranes	102 (36.7)	111 (38.0)	56 (37.3)	48 (39.7)	46 (35.9)	63 (36.8)
Chorioamnionitis, clinical	48 (17.3)	47 (16.1)	27 (18.0)	21 (17.4)	21 (16.4)	26 (15.2)
Antenatal steroids	278 (100.0)	292 (100.0)	150 (100.0)	121 (100.0)	128 (100.0)	171 (100.0)
Magnesium day of delivery	258 (92.8)	254 (87.0)	137 (91.3)	101 (83.5)	121 (94.5)	153 (89.5)
Cesarean delivery	186 (66.9)	207 (70.9)	111 (74.0)	98 (81.0)	75 (58.6)	109 (63.7)

Abbreviation: GA, gestational age.

^a^ American Indian or Alaska Native.

**Figure 1.**
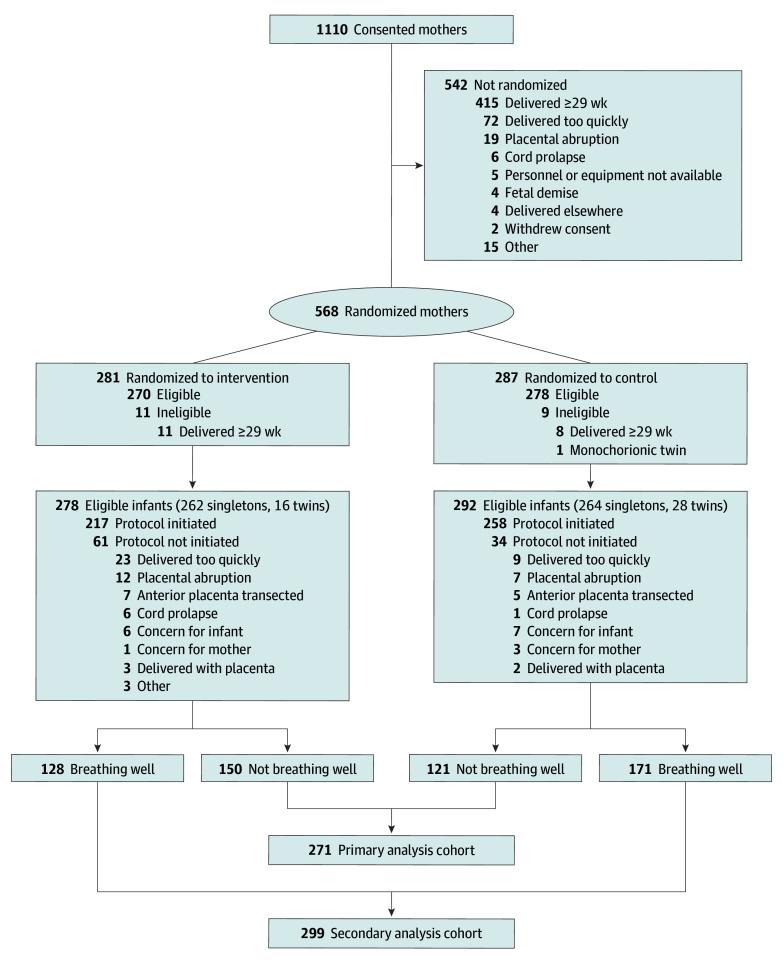
Patient Recruitment and Randomization

Consent was obtained from 1110 women admitted to the hospitals and anticipated to deliver at less than 29 weeks’ gestation (Figure 1). Of these, 568 women were randomized. The most common reasons for not randomizing were delivering at 29 weeks or later (415 [76.6%]) and delivering too quickly (72 [13.3%]). Nineteen women (3.3%) anticipated to deliver at less than 29 weeks were randomized but then delivered at 29 weeks or later and, therefore, were excluded from analysis. One monochorionic twin pregnancy was randomized in error; the protocol was not initiated, and this case was not included. Thus, there were 548 randomized mothers who delivered at less than 29 weeks, including 526 singleton and 22 twin births and 0 stillbirths. In total, 570 eligible infants were included in the analyses, with 278 randomized to the intervention and 292 to the control group.

Infant demographics and perinatal variables are shown in Table 1. The cohort of infants assessed as not breathing well 30 seconds after birth represented 47.5% of the total cohort (271 of 570; median [IQR] GA, 26.0 [24.7-27.4] weeks; 119 females [43.9%] and 152 males [56.1%]; maternal race: 2 Asian [1.7%], 77 Black [28.4%], 167 White [61.6%], 1 other [0.4%], 6 multiracial [2.2%], and 18 not reported [6.6%]; maternal ethnicity: 33 Hispanic or Latino [12.2%], 223 not Hispanic or Latino [82.3%], and 15 not reported [5.5%]), and those assessed as breathing well represented 52.5% (299 of 570; median [IQR] GA, 27.0 [25.7-28.0] weeks; 154 females [51.5%] and 145 males [48.5%]; maternal race: 14 Asian [4.7%], 92 Black [30.8%], 173 White [57.9%], 2 other [0.7%], 2 multiracial [0.7%], and 16 not reported [5.4%]; maternal ethnicity: 31 Hispanic or Latino [10.4%], 255 not Hispanic or Latino [85.3%], and 13 not reported [4.4%]). Maternal adverse events did not differ between groups (eTable1 in Supplement 2).

### Protocol Adherence

The protocol was initiated in 83.3% (475 of 570) of randomized infants, including 78.1% (217 of 278) in the intervention group and 88.4% (258 of 292) in the control group. The most common reasons for inability to initiate the protocol included placental emergencies in 6.3% (36 of 570, similar in the intervention and control groups) and precipitous deliveries with inadequate time for preparation, which occurred in 8.3% (23 of 278) of infants in the intervention group and 3.1% (9 of 292) in the control group.

Median cord clamping time, including cases in which the protocol was not initiated, was 120 seconds (IQR, 64-124 seconds) in the intervention group and 60 seconds (IQR, 30-63 seconds) in the control group. Immediate or early cord clamping less than 15 seconds after birth occurred in 15.1% (42 of 278) and 16.4% (48 of 292) of infants in the intervention and control groups, respectively.

### Primary Outcome

**Figure 2.**
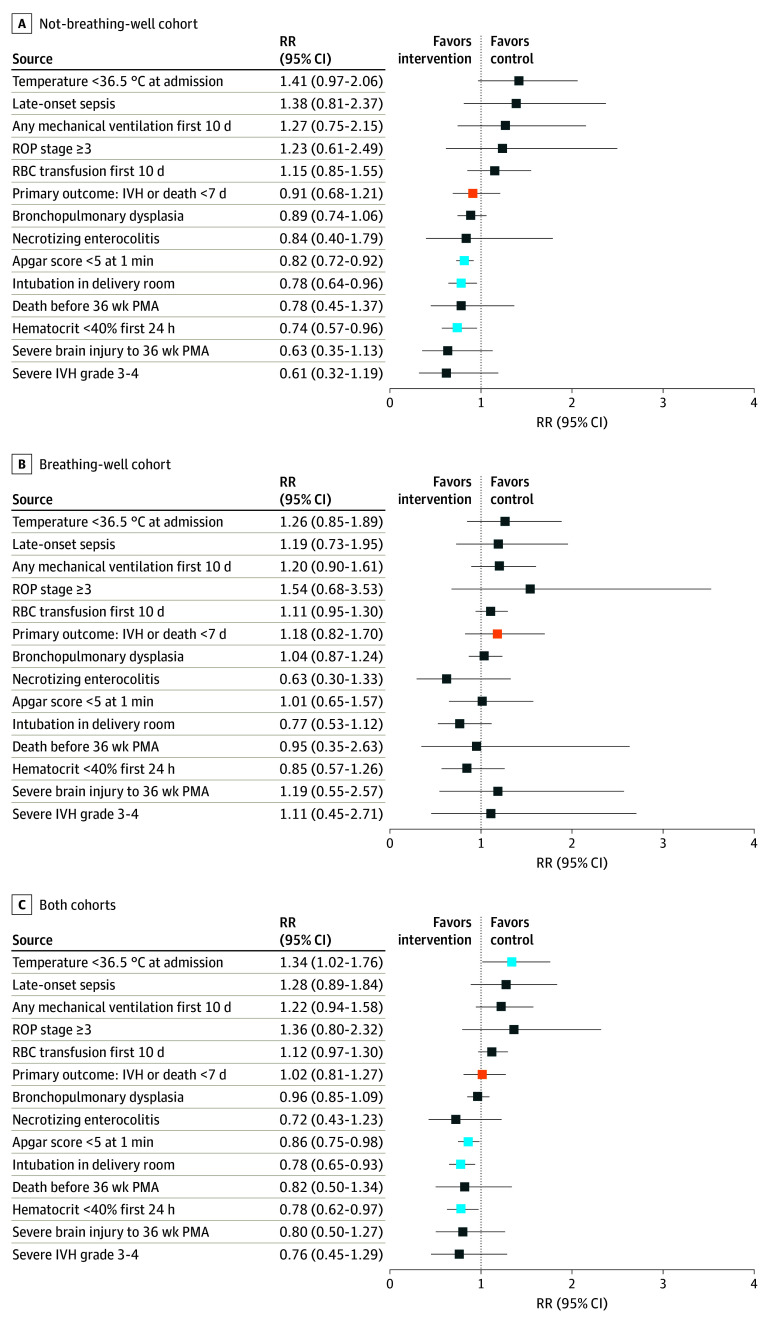
Relative Risk (RR) of Primary and Other Binary Outcomes in the Intervention (Intention to Treat) Compared With the Control Group, by Breathing Cohort Orange boxes indicate the primary outcome; light blue boxes indicate CIs that do not include 1; and dark blue boxes indicate CIs that include 1. IVH indicates intraventricular hemorrhage; PMA, postmenstrual age; RBC, red blood cell; ROP, retinopathy of prematurity.

In all 570 infants, IVH on 7- to 10-day head ultrasonography or death prior to day 7 occurred in 34.9% (97 of 278) of infants in the intervention group and 32.5% (95 of 292) of the control group (adjusted RR, 1.02; 95% CI, 0.81-1.27) (Figure 2). In the not-breathing-well cohort, IVH or death occurred in 38.7% (58 of 150) of infants in the intervention group and 43.0% (52 of 121) in the control group (RR, 0.91; 95% CI, 0.68-1.21). In the breathing-well cohort, IVH or death occurred in 30.5% (39 of 128) of infants in the intervention group and 25.1% (43 of 171) in the control group (RR, 1.18; 95% CI, 0.82-1.70).

### Secondary and Other Outcomes

Secondary outcomes are shown in Table 2. There was a difference in the median lowest hematocrit in the first 24 hours after birth between the intervention and control groups in the not-breathing-well cohort (median, 43% [IQR, 37%-47%] and 39% [IQR, 34%-43%], respectively). In the breathing-well cohort, the lowest hematocrit within 24 hours of birth was not different between groups. Other secondary outcomes were not different between groups in either breathing cohort.

**Table 2.** Primary and Secondary Outcomes by Randomization Before Birth

Outcome	Not breathing well at 30 s			Breathing well at 30 s		
	Intervention, No. (%) (n = 150)	Control, No. (%) (n = 121)	Statistic (95% CI)^a^	Intervention, No. (%) (n = 128)	Control, No. (%) (n = 171)	Statistic (95% CI)^a^
**Primary**						
IVH on 7-10-d HUS or death <7 d						
All GAs	58 (38.7)	52 (43.0)	RR, 0.91 (0.68 to 1.21)	39 (30.5)	43 (25.2)	RR, 1.18 (0.82 to 1.70)
GA 23 0/7 to 25 6/7 wk	32 (45.1)	30 (49.2)	RR, 0.92 (0.64 to 1.32)	22 (48.9)	18 (33.3)	RR, 1.47 (0.91 to 2.37)
GA 26 0/7 to 28 6/7 wk	26 (32.9)	22 (36.7)	RR, 0.90 (0.57 to 1.42)	17 (20.5)	25 (21.4)	RR, 0.96 (0.55 to 1.66)
Components of primary outcome						
IVH on 7-10-d HUS (survivors)	45 (30.0)	39 (32.2)	RR, 0.92 (0.65 to 1.29)	36 (28.1)	40 (23.4)	RR, 1.17 (0.80 to 1.72)
Death <7 d	13 (8.7)	13 (10.7)	RR, 1.05 (0.66 to 1.68)	3 (2.3)	3 (1.8)	RR, 1.47 (0.28 to 7.61)
**Secondary**						
Apgar score <5 at 5 min	27 (18.0)	25 (20.7)	RR, 0.89 (0.55 to 1.44)	5 (3.9)	5 (2.9)	RR, 1.30 (0.39 to 4.33)
Lowest hematocrit first 24 h, median (IQR), %^b^	43 (37-47)	39 (34-43)	MD, 3.34 (1.52 to 5.17)	44 (40 to 48)	44 (39 to 48)	MD, 0.73 (−0.91 to 2.37)
Medication for low blood pressure first 24 h^b^	25 (16.8)	28 (23.3)	RR, 0.74 (0.46 to 1.18)	8 (6.3)	14 (8.2)	RR, 0.74 (0.32 to 1.70)
No. of blood transfusions first 10 d, median (IQR)	1 (0-2)	1 (0-3)	MR, 0.85 (0.66 to 1.10)	0 (0 to 1)	0 (0 to 1)	MR, 1.01 (0.66 to 1.52)
Severe brain injury composite^c^	17 (11.3)	22 (18.2)	RR, 0.63 (0.35 to 1.13)	11 (8.6)	12 (7.0)	RR, 1.19 (0.55 to 2.57)
Death before 36 wk PMA	20 (13.3)	21 (17.4)	RR, 0.78 (0.45 to 1.37)	6 (4.7)	8 (4.7)	RR, 0.95 (0.35 to 2.63)

Abbreviations: GA, gestational age; HUS, head ultrasound; IVH, intraventricular hemorrhage; MD, mean difference; MR, mean ratio; PMA, postmenstrual age; RR, relative risk.

^a^ Cochran-Mantel-Haenszel RR (PROC FREQ) linear regression MD (PROC GLM); negative binomial regression MR (PROC GENMOD), SAS, version 9.4 (SAS Institute Inc).

^b^ Data not ascertained in nonsurvivors at relevant time points.

^c^ Grade 3 to 4 IVH, cystic periventricular leukomalacia or cerebellar hemorrhage by 36 weeks’ PMA.

Other outcomes of clinical interest are shown in eTable 2 in Supplement 2. Compared with the control group, infants randomized to the intervention group and assessed as not breathing well at 30 seconds had higher 1-minute Apgar scores (median, 3 [IQR, 2-5] vs 2 [IQR, 1-3], respectively) and were less likely to require endotracheal intubation in the delivery room (RR, 0.78; 95% CI, 0.64-0.96). Median NICU admission temperature was 0.2 °C lower in the intervention group (36.6 °C [IQR, 36.3 °C-36.9 °C] vs 36.8 °C [IQR, 36.5 °C-37.1 °C] in the control group) in the not-breathing-well cohort. There was no difference in admission temperature in the breathing-well cohort.

The forest plot in Figure 2 shows the RRs of the primary outcome and select binary outcomes. For the intervention group compared with the control group, the RRs of Apgar score less than 5 at 1 minute and hematocrit less than 40% in the first 24 hours were less than 1, with 95% CIs excluding 1 in the not-breathing-well cohort (0.82 [95% CI, 0.72-0.92] and 0.74 [95% CI, 0.57-0.96], respectively) and the combined breathing cohorts (0.86 [95% CI, 0.75-0.98] and 0.78 [95% CI, 0.62-0.97], respectively) but not in the breathing-well cohort (1.01 [95% CI, 0.65-1.57] and 0.85 [95% CI, 0.57-1.26], respectively).

### Additional Analyses

Adjustment for female sex in the primary outcome resulted in an RR of 0.98 (95% CI, 0.70-1.37) and for male sex, 1.14 (95% CI, 0.83-1.54). A sensitivity analysis was performed as described in eMethods 1 in Supplement 2. Results support potential bias in breathing assessment (eFigure 1A in Supplement 2) but with no or minimal effect on the outcomes assessment (eFigure 1B-F in Supplement 2). Additional analyses included twin correlation (eMethods 2 in Supplement 2), per-protocol (eFigure 2 in Supplement 2), and site-adjusted intention-to-treat (eFigure 3 in Supplement 2) outcomes, none of which substantially altered study findings.

## Discussion

In this randomized clinical trial of extremely preterm infants, providing assisted ventilation starting 30 seconds after delivery and clamping the umbilical cord at 120 seconds, compared with 30 to 60 seconds of delayed cord clamping followed by assisted ventilation, did not reduce the primary outcome of IVH at age 7 to 10 days or death prior to day 7. There were no discernable differences in mortality, severe brain injury, or common morbidities of prematurity through 36 weeks’ PMA.

We designed the VentFirst study to focus on extremely preterm infants not breathing well shortly after birth, a population excluded from many prior randomized clinical trials.^5,6,8^ Improved lung aeration with delivery of PPV in the not-breathing-well cohort may have been responsible for greater placental transfusion^19^ and more stable transition, but we did not assess the degree of lung aeration with our study design. We also did not assess other physiologic perturbations in the intrapartum and postpartum periods, which may have contributed to the primary outcome of IVH or early mortality.^20^ We did, however, show that providing assisted ventilation prior to cord clamping was feasible and safe. The protocol was initiated for 83.3% of infants randomized before birth to the intervention, and the only identified safety concern was mild hypothermia.

We found that the lowest hematocrit on the first day after birth was higher in the intervention compared with the control group in the cohort of infants not breathing well at 30 seconds but not in those breathing well, suggesting no substantial difference in placental transfusion in the latter group. There was no difference in number of blood transfusions within 10 days of birth in either cohort. This finding is consistent with results from a single-center randomized clinical trial of extremely preterm infants not breathing well 15 seconds after birth, in which providing assisted ventilation during 50 seconds of delayed cord clamping compared with the same duration of delayed clamping followed by assisted ventilation did not reduce the need for blood transfusion.^12^

Among infants assessed as not breathing well at 30 seconds, we found that those randomized to the intervention had a higher Apgar score at 1 minute and were less likely to have endotracheal intubation in the delivery room compared with infants in the control group. Since clinicians at the delivery could not be masked to group assignment, there could have been bias in Apgar score assignment or decision for intubation, but it is also possible that providing assisted ventilation prior to cord clamping led to improved stability in the delivery room. Aside from these differences, other clinical outcomes were not notably different, which may reflect that death and major morbidities associated with extremely preterm birth are complex, multifactorial, and affected by many factors other than provision of assisted ventilation during delayed cord clamping.

### Limitations

There are several limitations to consider in interpreting results of this trial and considering the design of future trials. Antenatal consent was required but often not feasible; thus, the study population does not fully represent all extremely preterm births. Delivery room clinicians were aware of study arm, potentially introducing bias for breathing assessment but likely not influencing study conclusions. An additional limitation is that we could not assess lung aeration prior to cord clamping. In another trial of physiologic-based cord clamping, respiratory and vital sign monitoring during the intervention were used to determine when to cut the cord.^21^

## Conclusions

In this multicenter, randomized clinical trial of extremely preterm infants, ventilatory assistance starting at 30 seconds followed by cord clamping at 120 seconds, compared with cord clamping at 30 to 60 seconds after birth followed by ventilatory assistance, did not affect the occurrence of any grade of IVH by age 7 to 10 days or death before day 7. Given the importance of remaining issues around feasibility, safety, and efficacy of providing assisted ventilation to extremely preterm infants before cord clamping, and the difficulty in conducting large randomized clinical trials in this population, individual participant data meta-analysis of similarly designed trials, as planned by the Individual Participant Data on Cord Management at Preterm Birth Collaboration,^6^ may provide additional insights.

## Notes

Supplement 1.
Trial Protocol, Protocol Amendments, and Statistical Analysis Plan

Supplement 2. **eTable 1.** Maternal Adverse Events **eTable 2.** Other Outcomes **eMethods 1.** Sensitivity Analysis: Bias in Not-Breathing-Well Cohort Assessment **eFigure 1.** Sensitivity Analysis for Bias in the Not-Breathing-Well Cohort Assessment **eMethods 2.** Sensitivity Analysis: Twin Correlations **eFigure 2.** Per-Protocol Analysis of Primary and Select Binary Outcomes **eFigure 3.** Site-Adjusted Intention-to-Treat Analysis

Supplement 3.
Nonauthor Collaborators

Supplement 4.
Data Sharing Statement
