# Supplementary material for: Ventilatory Assistance Before Umbilical Cord Clamping in Extremely Preterm Infants: A Randomized Clinical Trial
Source: JAMA Netw Open. 2024 May 17;7(5):e2411140. doi: 10.1001/jamanetworkopen.2024.11140 (PMC11102017; doi:10.1001/jamanetworkopen.2024.11140)
Supplement: Supplement 2. — eTable 1. Maternal Adverse Events eTable 2. Other Outcomes eMethods 1. Sensitivity Analysis: Bias in Not-Breathing-Well Cohort Assessment eFigure 1. Sensitivity Analysis for Bias in the Not-Breathing-Well Cohort Assessment eMethods 2. Sensitivity Analysis: Twin Correlations eFigure 2. Per-Protocol Analysis of Primary and Select Binary Outcomes eFigure 3. Site-Adjusted Intention-to-Treat Analysis [file jamanetwopen-e2411140-s002.pdf]

Supplemental Online Content

Fairchild KD, Petroni GR, Varhegyi NE, et al. Ventilatory assistance before umbilical cord clamping in extremely preterm infants: a randomized clinical trial. *JAMA Netw Open*. 2024;7(5):e2411140. doi:10.1001/jamanetworkopen.2024.11140

eTable 1. Maternal Adverse Events

eTable 2. Other Outcomes

eMethods 1. Sensitivity Analysis: Bias in Not-Breathing-Well Cohort Assessment

eFigure 1. Sensitivity Analysis for Bias in the Not-Breathing-Well Cohort Assessment

eMethods 2. Sensitivity Analysis: Twin Correlations

eFigure 2. Per-Protocol Analysis of Primary and Select Binary Outcomes

eFigure 3. Site-Adjusted Intention-to-Treat Analysis

This supplemental material has been provided by the authors to give readers additional information about their work.

© 2024 Fairchild KD et al. *JAMA Network Open.*

eTable 1. Maternal Adverse Events

	Total n=548	Intervention n=270	Control n=278
Postpartum hemorrhage ≥1000 ml[a]	78 (14.2)	40 (14.8)	38 (13.7)
Retained placenta[b]	14 (2.6)	7 (2.6)	7 (2.5)
Maternal blood transfusion[b]	23 (4.2)	7 (2.6)	16 (5.8)
Postpartum delivery-related infection[b]	6 (1.1)	3 (1.1)	3 (1.1)

Results presented as n (%)
[a] within 24 hours of delivery
[b] prior to discharge

© 2024 Fairchild KD et al. *JAMA Network Open.*

eTable 2. Other Outcomes

Randomization before birth		Intervention	Control		Intervention	Control	
Breathing cohort at 30 seconds	# % ascertained[a]	*Not breathing well*			*Breathing well*		
		n=150	n=121	Stat (95% CI)[b]	n=128	n=171	Stat (95% CI)[b]
**Delivery Room**							
Cord clamp time (seconds from birth)	569<br>99.8	105 (20, 122)	30 (5,35)	NA	120 (119, 125)	62 (60, 66)	NA
Umbilical artery pH	397<br>70.0	7.2 (7.1, 7.3)	7.3 (7.2, 7.3)	MD -0.02 (-0.05, 0.01)	7.3 (7.2, 7.3)	7.3 (7.2, 7.3)	MD 0.00 (-0.02, 0.03)
Apgar 1 minute	570<br>100.0	3 (2, 5)	2 (1, 3)	**MD 0.98 (0.56, 1.40)**	6 (5, 7)	6 (5, 7)	MD 0.02 (-0.38, 0.42)
Apgar 5 minutes	570<br>100.0	7 (5, 8)	6 (5, 7)	MD 0.20 (-0.24, 0.64)	8 (7, 8)	8 (7, 8)	MD -0.01 (-0.31, 0.30)
Intubation in delivery room	570<br>100.0	71 (47.3)	75 (62.0)	**RR 0.78 (0.64, 0.96)**	28 (21.9)	46 (26.9)	RR 0.77 (0.53, 1.12)
**NICU first 24 hours**							
NICU admission temp. °C	567<br>99.5	36.6 (36.3, 36.9)	36.8 (36.5, 37.1)	**MD -0.21 (-0.36, -0.06)**	36.8 (36.4, 37.1)	36.8 (36.5, 37.1)	MD -0.10 (-0.23, 0.03)
Lowest mean blood pressure (mmHg)	567<br>99.5	24 (21, 28)	22 (19, 26)	MD 1.62 (0.41, 2.38)	26 (22, 30)	26 (22, 30)	MD 0.39 (-1.07, 1.85)
Volume infusion	570<br>100.0	72 (48.0%)	65 (53.7%)	RR 0.90 (0.72, 1.14)	39 (30.5%)	46 (26.9%)	RR 1.11 (0.78, 1.59)
Pneumothorax treated	568<br>99.7	1 (0.7%)	4 (3.3%)	RR 0.19 (0.02, 1.72)	2 (1.6%)	2 (1.2%)	RR 1.29 (0.20, 8.46)
Highest hematocrit	565<br>99.1	46 (41, 49)	42 (37, 47)	MD 2.45 (0.62, 4.28)	48 (44, 52)	46 (41, 49)	MD 1.87 (0.23, 3.51)
SNAPPE-II score	422<br>74.0	43 (27, 59)	49 (31, 61)	MD -3.11 (-8.67, 2.46)	31 (14, 42)	27 (15, 42)	MD -0.50 (-4.89, 3.89)
**NICU first 10 days**							
Days on mechanical ventilation	565<br>99.1	4.5 (1, 10)	5 (2, 10)	MR 0.90 (0.73, 1.11)	1 (0, 6.5)	2 (0, 7)	MR 0.92 (0.67, 1.27)
Early onset sepsis	567<br>99.5	8 (5.4%)	5 (4.2%)	RR 1.27 (0.43, 3.76)	5 (3.9%)	4 (2.3%)	RR 1.66 (0.46, 5.97)
Maximum serum bilirubin (mg/dl)	563<br>98.8	6.5 (5.3, 7.8)	6.3 (5.7, 7.8)	MD -0.29 (-0.72, 0.14)	7 (6, 8.1)	6.7 (5.7, 8.1)	MD 0.16 (-0.21, 0.53)
Days of phototherapy	564<br>98.9	5 (3, 6)	5 (3, 7)	MR 0.96 (0.86, 1.07)	5 (3, 6)	5 (3, 6)	MR 1.07 (0.96, 1.19)

Randomization before birth		Intervention	Control		Intervention	Control	
**NICU through 36 weeks PMA**							
Late onset sepsis	542<br>95.1	28 (20.4%)	17 (15.2%)	RR 1.38 (0.81, 2.37)	25 (19.8%)	27 (16.2%)	RR 1.19 (0.73, 1.95)
PDA treated	538<br>94.4	36 (26.5%)	35 (31.5%)	RR 0.87 (0.60, 1.27)	32 (25.8%)	46 (27.5%)	RR 0.90 (0.62, 1.31)
Spontaneous intestinal perforation	538<br>94.4	7 (5.2%)	5 (4.5%)	RR 1.18 (0.39, 3.58)	4 (3.2%)	3 (1.8%)	RR 1.71 (0.40, 7.36)
Necrotizing enterocolitis	539<br>94.6	12 (8.8%)	12 (10.8%)	RR 0.84 (0.40, 1.79)	9 (7.2%)	18 (10.8%)	RR 0.63 (0.30, 1.33)
ROP stage ≥3	535<br>93.9	15 (11.2%)	11 (9.9%)	RR 1.23 (0.61, 2.49)	11 (8.9%)	9 (5.4%)	RR 1.54 (0.68, 3.53)
Bronchopulmonary dysplasia	523<br>91.8	80 (61.1%)	74 (69.8%)	RR 0.89 (0.74, 1.06)	77 (63.6%)	100 (60.6%)	RR 1.04 (0.87, 1.24)
Maximum grade IVH	549[c]<br>96.3						
0		83 (59.7%)	66 (57.4%)		83 (65.9%)	118 (69.8%)	
1		32 (23.0%)	20 (17.4%)		23 (18.3%)	33 (19.5%)	
2		11 (7.9%)	11 (9.6%)		12 (9.5%)	9 (5.3%)	
3		5 (3.6%)	2 (1.7%)		1 (0.8%)	2 (1.2%)	
4		8 (5.8%)	16 (13.4%)		7 (5.6%)	7 (4.1%)	

Results presented as n (%) or median (25th, 75th %ile)
[a] Most common reason for non-ascertainment is non-survival
[b] Cochran Mantel Haenszel RR relative risk, GLM MD mean difference, GENMOD MR mean ratio
[c] Infants who died at <7 days of age not included
Bolded values are statistically significantly different in intervention versus control groups
IVH = intraventricular hemorrhage, PDA = patent ductus arteriosus, PMA = post-menstrual age, ROP = retinopathy of prematurity. SNAPPE-II = Score for Neonatal Acute Physiology-Perinatal Extension-II

© 2024 Fairchild KD et al. *JAMA Network Open.*

eMethods 1. Sensitivity Analysis: Bias in not-breathing-well cohort assessment

Purpose: There was arm imbalance in the breathing cohorts, with 54% in the intervention arm assessed as not breathing well within 30 seconds after birth versus 41% in controls. Randomization occurred before delivery, and clinicians assessing whether or not infants were breathing well could not be masked and may have been biased toward providing positive-pressure ventilation for infants randomized to the intervention. We sought to assess potential bias in breathing classification and its impact on study outcome analyses.

Methods: Using data from infants randomized to the control arm we developed a model to predict which infants were assessed as not breathing well within 30 seconds after birth. Model prediction was based on maternal parameters (preterm prelabor rupture of membranes, clinical chorioamnionitis, antepartum magnesium, Cesarean delivery) and newborn parameters (gestational age, birth weight, twin, sex, race). The model was applied to the data for the intervention group and a calibration curve was constructed to assess for bias in the assessment of not breathing well. We then assessed the potential impact of bias on study outcomes. Cochran-Mantel-Haenszel Relative Risk (CMH RR) and 95% confidence interval (CI) estimations were re-run sequentially deleting intervention infants with the lowest model-predicted probability of being assessed as not breathing well, until there were equal numbers not-breathing-well cohort infants in the control and intervention groups. With this strategy, data from 29 intervention infants were sequentially deleted.

© 2024 Fairchild KD et al. *JAMA Network Open.*

Results: eFigure 1A displays the model calibration curve showing good calibration for control infants (black) and suggesting bias in the assessment for not-breathing-well cohort assignment for infants randomized to the intervention (blue). In eFigure 1B-F, relative risk and 95% CI from the original analyses are shown on the far left in orange. Parameter estimation results after sequential deletion of data from 29 infants at lowest model-assessed risk of not breathing well are shown for 5 outcomes, including the primary study outcome (1B) and 4 outcomes of possible interest found in the original intention-to-treat analysis: One minute Apgar score <5 (1C), intubation in the delivery room (1D), hematocrit <40 within 24 hours of birth (1E), and NICU admission temperature <36.5ºC (1F).

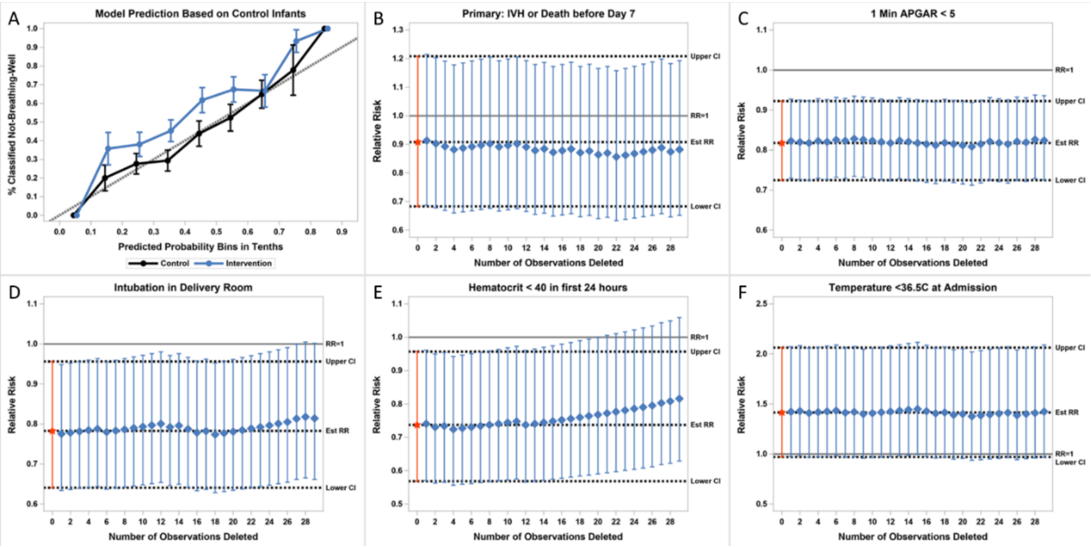

eFigure1. Sensitivity Analysis for Bias in the Not-Breathing-Well Cohort Assessment. (A) Model calibration curve for control (black) and intervention (blue) groups. (B-F) Sequential deletion plots showing the RR and CI from the original analysis (orange, far left) and the RR and CI after data from each of 29 infants with model-predicted lower risk for not breathing well were removed from the analysis (blue). Dotted lines display lower CI, RR, and upper CI from the original analysis. Solid grey line represents RR=1.

© 2024 Fairchild KD et al. *JAMA Network Open.*

Conclusion: This analysis indicates there may have been bias in not-breathing-well cohort assessment, but this did not impact study conclusions.

eMethods 2. Sensitivity Analysis: Twin correlation

Purpose: Of the 570 infants in the study, there were 22 sets of twins (44 infants, or 7.7% of the study cohort). Breathing assessment differed in 14 sets of twins, with 8 first-born twins assessed as not breathing well and 6 as breathing well. In the not-breathing-well cohort, 5 sets or 10 infants were twins (3.7%).

Method: Generalized estimating equations were used to account for any correlation between twins in the primary outcome.

Result: Accounting for this low level of twins resulted in relative risk estimation for the primary outcome in the not-breathing-well cohort of 0.904 (95% CI 0.68, 1.21).

Conclusion: This was a minimal difference when compared to the primary analysis result of 0.908 (95% CI 0.68, 1.21).

© 2024 Fairchild KD et al. *JAMA Network Open.*

eFigure2. Per-Protocol Analysis for Primary and Select Binary Outcomes. Per-protocol analyses included all infants except those for whom the protocol could not be initiated or for whom cord clamping occurred more than 15 seconds from the protocol-prescribed time, for a total n=413. Cochran-Mantel-Haenszel Relative Risk and 95% confidence interval (CI) estimates adjusted for GA are shown based on breathing cohort (not-breathing-well left; breathing-well center) and for the combined cohorts (right). The orange star and bars show RR and CI of the primary outcome of any grade IVH on 7-10 day head ultrasound or death before day 7. The blue diamonds and bars indicate outcomes for which the CI bound does not cross 1. ROP=retinopathy of prematurity; RBC-red blood cell; IVH=intraventricular hemorrhage.

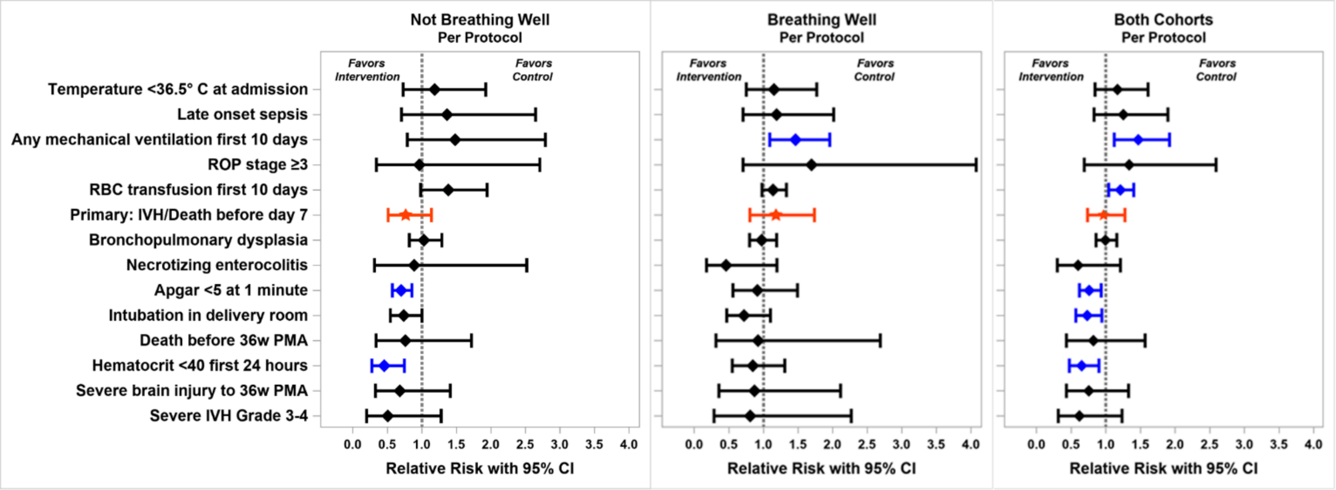

© 2024 Fairchild KD et al. *JAMA Network Open.*

eFigure3. Site-Adjusted Intention-to-Treat Analysis. Cochran-Mantel-Haenszel Relative Risk and 95% confidence interval (CI) estimates adjusted for GA and Site are shown. Sites with <25 enrolled infants were grouped as one. Orange star and bars represent RR and CI of the primary outcome of any grade IVH or death before day 7. Blue diamonds and bars indicate outcomes for which the CI bound does not cross 1. ROP=retinopathy of prematurity; RBC-red blood cell; IVH=intraventricular hemorrhage.

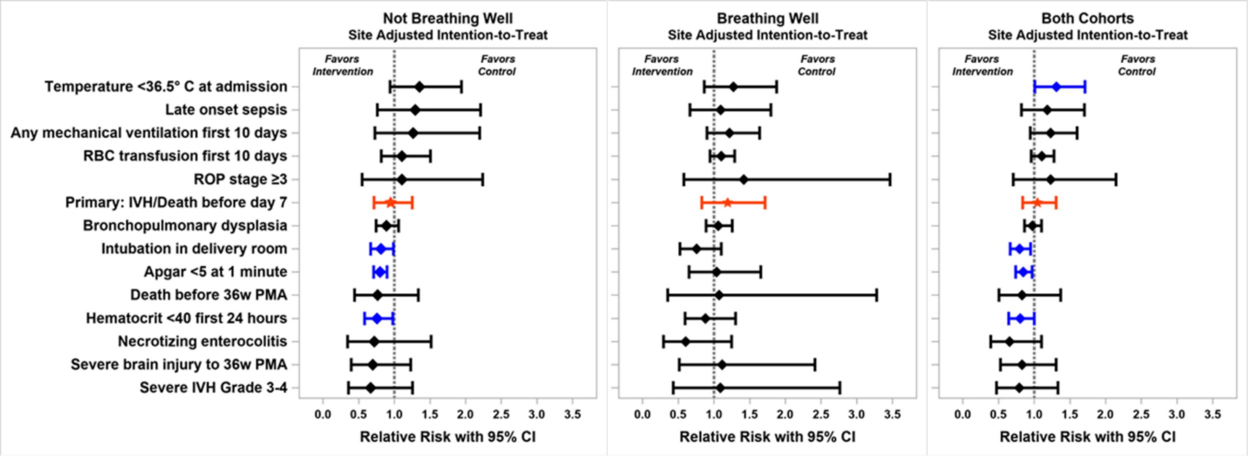

© 2024 Fairchild KD et al. *JAMA Network Open.*
