# Supplementary material for: Ventilatory Assistance Before Umbilical Cord Clamping in Extremely Preterm Infants: A Randomized Clinical Trial
Source: JAMA Netw Open. 2024 May 17;7(5):e2411140. doi: 10.1001/jamanetworkopen.2024.11140 (PMC11102017; doi:10.1001/jamanetworkopen.2024.11140)
Supplement: Supplement 3. — Nonauthor Collaborators [file jamanetwopen-e2411140-s003.pdf]

Supplemental Online Content: Nonauthor Collaborators
*First name, last name, and suffix (if applicable) are required and will appear in PubMed.

*Group Name(s): VentFirst Consortium							
***First Name and Middle Initial(s)**	***Last Name**	***Suffix (eg, Jr, III)**	Academic Degrees	Institution	Location (city, state/province, country)	Role or Contribution, eg, chair, principal investigator	Group (if more than 1 Group listed in the byline) and/or Subgroup (eg, Steering Committee)
Gina M	Duda		BS	University of Virginia School of Medicine	Charlottesville, VA, USA	Clinical Research Coordinator	
Monika	Thielen		ART	University of Virginia School of Medicine	Charlottesville, VA, USA	Clinical Research Coordinator	
Lavonne M	Liedl		RRT	Mayo Clinic College of Medicine and Science	Rochester, MN, USA	Clinical Research Coordinator	
Amy L	Amsbaugh		RRT	Mayo Clinic College of Medicine and Science	Rochester, MN, USA	Clinical Research Coordinator	
Amy	Lamprecht		BSN	University of Colorado School of Medicine	Aurora, CO, US	Clinical Research Coordinator	
Carolyn S	Berlinski		BSN	University of Colorado School of Medicine	Aurora, CO, US	Clinical Research Coordinator	
Melissa	Hawkins		RN	Saint Louis University School of Medicine	St. Louis, MO, USA	Clinical Research Coordinator	
Jacque-Lynne F	Johnson		PhD	University of Calgary	Calgary, AB, Canada	Clinical Research Coordinator	
Leigh	Irvine		MN	University of Calgary	Calgary, AB, Canada	Clinical Research Coordinator	
Anna-Maria	Ciorogariu-Ivan		BSc	University of Calgary	Calgary, AB, Canada	Clinical Research Coordinator	
Rosa R	Pesavento		BA	University of California, Davis School of Medicine	Sacramento, CA, USA	Clinical Research Coordinator	
Caroline	Fray		BSN	University of Alberta, Edmonton	Edmonton, AB, Canada	Clinical Research Coordinator	
Melba	Athaide		BSN	University of Alberta, Edmonton	Edmonton, AB, Canada	Clinical Research Coordinator	
