# Supplementary material for: Ventilatory Assistance Before Umbilical Cord Clamping in Extremely Preterm Infants: A Randomized Clinical Trial
Source: JAMA Netw Open. 2024 May 17;7(5):e2411140. doi: 10.1001/jamanetworkopen.2024.11140 (PMC11102017; doi:10.1001/jamanetworkopen.2024.11140)
Supplement: Supplement 4. — Data Sharing Statement [file jamanetwopen-e2411140-s004.pdf]

# Data Sharing Statement

Fairchild. Ventilatory Assistance Before Umbilical Cord Clamping in Extremely Preterm Infants. *JAMA Netw Open*. Published May 17, 2024. doi:10.1001/jamanetworkopen.2024.11140

**Data**
**Data available:** Yes
**Data types:** Deidentified participant data
**How to access data:** https://www.icompstudy.org/
**When available:** beginning date: 07-01-2024

**Supporting Documents**
**Document types:** None

**Additional Information**
**Who can access the data:** iCOMP consortium
**Types of analyses:** IPD metaanalysis
**Mechanisms of data availability:** data will be available to the iCOMP group for IPD MA which will be published
